# Visual Acuity and Associated Factors. The Central India Eye and Medical Study

**DOI:** 10.1371/journal.pone.0022756

**Published:** 2011-08-04

**Authors:** Vinay Nangia, Jost B. Jonas, Ajit Sinha, Rajesh Gupta, Shubhra Agarwal

**Affiliations:** 1 Suraj Eye Institute, Nagpur, Maharashtra, India; 2 Department of Ophthalmology, Faculty of Clinical Medicine Mannheim, University of Heidelberg, Mannheim, Germany; Washington University School of Medicine, United States of America

## Abstract

Visual acuity is a major parameter for quality of vision and quality of life. Information on visual acuity and its associated factors in rural societies almost untouched by any industrialization is mostly non-available. It was, therefore, the purpose of our study to determine the distribution of visual acuity and its associated factors in a rural population not marked influenced by modern lifestyle. The population-based Central India Eye and Medical Study included 4711 subjects (aged 30+ years), who underwent a detailed ophthalmologic examination including visual acuity measurement. Visual acuity measurements were available for 4706 subjects with a mean age of 49.5±13.4 years (range: 30–100 years). BCVA decreased significantly (*P*<0.001) from the moderately hyperopic group (0.08±0.15 logMAR) to the emmetropic group (0.16±0.52 logMAR), the moderately myopic group (0.28±0.33 logMAR), the highly hyperopic group (0.66±0.62 logMAR) and finally the highly myopic group (1.32±0.92 logMAR). In multivariate analysis, BCVA was significantly associated with the systemic parameters of lower age (*P*<0.001), higher level of education (*P*<0.001), higher body stature (*P*<0.001) and higher body mass index (*P*<0.001), and with the ophthalmic parameters of more hyperopic refractive error (spherical equivalent) (*P*<0.001), shorter axial length (*P*<0.001), lower degree of nuclear cataract (*P*<0.001), and lower intraocular pressure (*P* = 0.006). The results suggest that in the rural population of Central India, major determinants of visual acuity were socioeconomic background, body stature and body mass index, age, refractive error, cataract and intraocular pressure.

## Introduction

Visual acuity is the major parameter for quality of vision and it is of utmost importance for quality of life. Recent population-based studies on various ethnic and socio-cultural groups have examined the prevalence and associated factors of low vision and blindness at the low end of the visual acuity scale [1]–[5]. Relatively few epidemiological investigations have addressed the distribution and the associated factors of the whole range of visual acuity. This information is, however, interesting since it may show which ocular, systemic and general parameters including socioeconomic variables play a role for visual acuity, and indirectly for quality of vision. We, therefore, conducted the present study to examine the distribution of visual acuity in a population-based setting. We chose a rural population in Central India, since it additionally opened the possibility to address the study questions in a society not markedly influenced by modern lifestyle.

## Materials and Methods

### Ethics Statement

The Medical Ethics Committee of the Medical Faculty Mannheim of the Ruprecht-Karls-University Heidelberg and the ethical committee of Suraj Eye Institute / Nagpur approved the study and all participants gave informed written consent.

The Central India Eye and Medical Study (CIEMS) is a population-based cross-sectional study in Central India. It was carried out in 8 villages in the rural region of Central Maharashtra at a distance of about 40 km from Nagpur [6], [7]. Inclusion criterion was an age of 30+ years. The villages were chosen for the study since they were located in a typically rural region of Central India at a relatively large distance to the next city (Nagpur). The Suraj Eye Institute was already known to the villagers, and the villagers had attended the institute for cataract surgery for the last 10 years. In a first step, social workers mapped the villages and went from house to house to list all inhabitants. They invited all villagers with an age of 30+ years to participate in the study including a bus ride to the hospital, a one-day long examination, meals, and a return bus ride to the village in the evening all at no cost. Up to 20 subjects were examined per day. If cataract was diagnosed, cataract and intraocular lens implant surgery was offered free of charge to the patients. The recruitment of the study participants took place between the mid of 2006 and mid of 2008.

Out of total population of 13,606 villagers, 5885 subjects fulfilled the inclusion criterion of an age of 30+ years and were eligible for the study. Out of these 5885 subjects, 4711 people participated, resulting in a response rate of 80.1%. There were 2520 (53.5%) women. All examinations were carried out in the hospital. Trained social workers filled out a questionnaire including questions on the socioeconomic background (such as profession, monthly family income, possession of a mobile) and living conditions (such as toilet available in the house, lighting source, agricultural land and livestock ownership, size of family), on the daily food (such as vegetables, fruits, meat, how often per day and how many servings), smoking or other types of tobacco consumption, alcohol consumption, amount and type of daily physical activity, known diagnosis of diabetes mellitus, arterial hypertension, bronchial asthma, angina pectoris or other heart problems, cancer, diseases of the thyroid gland, malaria and tuberculosis), on the intake of medication such as aspirin and steroids, on the psychiatric status (questions on psychiatric depression including thoughts of suicide), on wearing and availability of glasses, and on the family history of eye diseases.

The ophthalmic examinations started with testing of visual acuity by ophthalmologists or optometrists. Uncorrected visual acuity, and visual acuity with the subjects' glasses and after refractive correction was measured using modified ETDRS charts (Light House Low Vision Products, New York, NY, USA) at a distance of 4 meters. Automated refractometry and subjective refraction was performed for all subjects independent of the visual acuity. Keratometry was performed using a non-automatic keratometer (Appassawamy Ass., Chennai, India). Visual field examinations were performed with frequency-doubling perimetry using the screening program C-20-1 (Zeiss-Humphrey, Dublin, California, USA). Slit lamp biomicroscopy was carried out by a fellowship trained ophthalmologist and any abnormality in the anterior segment was noted. Intraocular pressure was measured by a slit lamp mounted Goldmann applanation tonometer. If the measurements were higher than 21 mm Hg, tonometry was repeated. With the subject in the supine position, ocular pachymetry and biometry were carried out with sonography using the Pacscan (Sonomed, U.S.A). Central corneal thickness, anterior chamber depth, lens thickness and axial length were measured for both eyes of all subjects. The pupil was dilated using tropicamide and phenylephrine 5% thrice at 15 minutes intervals for all subjects to attain the maximal pupillary dilatation. Digital photographs of the lens according to the Age Related Eye Disease Study for nuclear sclerosis grading were taken [8]. Retro-illuminated photographs of the lens for assessment of the cortical opacities were obtained using the ZeisFF450 telecentric fundus camera (Zeiss Meditec Co. Oberkochen, Germany). Digital monoscopic photographs of the optic disc (20 degree) and of the disc and macula (50 degree) were also taken. The magnification by the optic media of the eye were corrected by the inbuilt algorithm.

Selection criteria for the present study were the availability of measurements of visual acuity, i.e., we included all study participants with visual acuity measurements, independently, whether there was a reduction in visual acuity due to reasons that were independent of the parameters which were examined in this study (e.g. trauma, diabetic retinopathy, etc). Statistical analysis was performed using a commercially available statistical software package (SPSS for Windows, version 17.0, SPSS, Chicago, IL). In a first step, we examined the mean values (presented as mean ± standard error for frequency parameters; presented as mean ± standard deviation for all other parameters) of the systemic and ocular parameters. Visual acuity measurements were presented as the negative value of the decadal logarithm of the angle of minimal resolution (logMAR) and as decimal. In a second step, multivariate regression analyses were performed including those parameters as independent parameters which were significantly associated with the dependent parameter in univariate analysis. Chi-square tests were used to compare proportions. 95% Confidence intervals (CI) and odds ratios (OR) were presented. All *P*-values were 2-sided and were considered statistically significant when the values were less than 0.05. Unless an intra-individual comparison was performed, only one randomly selected eye per subject was taken for statistical analysis.

## Results

In the whole study population, the mean age was 49.5±13.4 years (median: 47 years; range: 30–100 years). The mean reported monthly income was 1584±1233 Rupee (median: 1350 Rupee; range: 200 to 15,000 Rupee; 1 US$ = about 50 Rupee). Out of the total 4711 subjects, 1623 (34.5%) subjects reported to be illiterate, 1310 (27.8%) subjects to have visited school up the 5th standard, 533 (11.3%) subjects to have visited school between the 6th to 8th standard, 1070 (22.7%) subjects to have attended school between the 9th and 12th standard, and 165 (3.5%) subjects reported to have received a higher level of education such as graduation or others. Ten (0.2%) subjects did not describe their level of education. Out of the 4711 study participants, visual acuity measurements were available for 9411 (99.9%) eyes of 4706 (99.9%) individuals out of the 4711 subjects participating in the study. The 5 (0.1%) subjects without visual acuity measurements were not cooperative for determination of visual acuity, due to reasons such as mental retardation.

### Univariate Analysis

**Figure 1:**
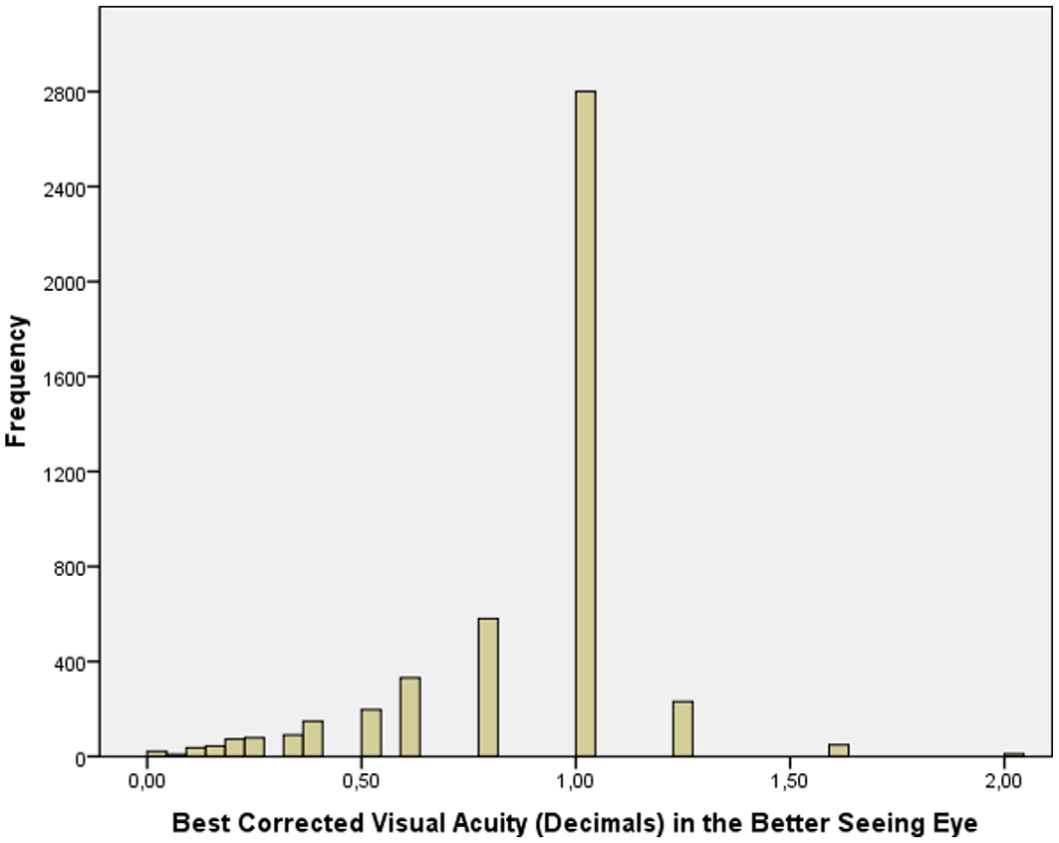
Histogram showing the distribution of best corrected visual acuity in adult Indians in the Central India Eye and Medical Study.

Taking the whole study population and including one randomly selected eye per subject into the statistical analysis, presenting visual acuity measured 0.33±0.49 logMAR (median: 0.00 logMAR; range: −0.30 to 3.00 logMAR) or 0.66±0.38 (expressed in decimal) (median: 0.79; range: 0.00 to 2.00). The mean best corrected visual acuity measured 0.17±0.43 logMAR (median: 0.00 logMAR; range: −0.30 to 3.00 logMAR) or 0.82±0.31 (expressed in decimal) (median: 1.00; range: 0.00 to 2.00). If the better seeing eye of each study participant was included into the statistical analysis, mean best corrected visual acuity was 0.10±0.24 logMAR (median: 0.00 logMAR; range: −0.30 to 2.70 logMAR) or 0.87±0.28 (expressed in decimal) (median: 1.00; range: 0.00 to 2.00) (Fig. 1).

**Figure 2:**
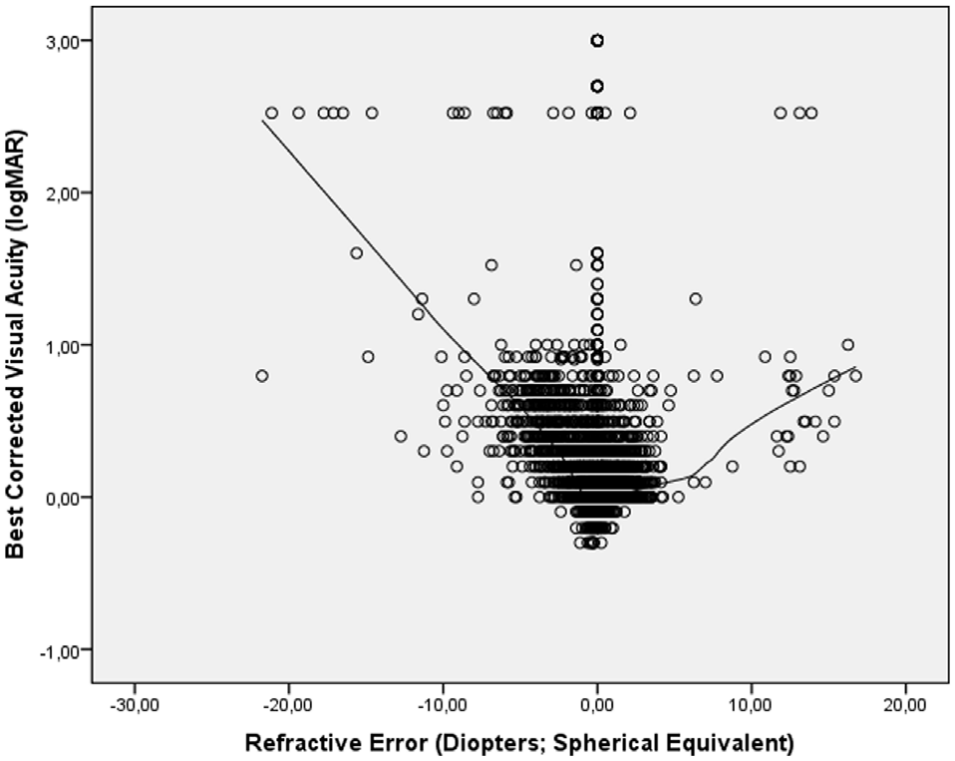
Scattergram showing the relationship between best corrected visual acuity (expressed as the logarithm of the minimal angle of resolution, logMAR) and refractive error in the Central India Eye and Medical Study; the regression line was calculated using a locally weighted scatterplot smoothing (LOWESS) function. The subgroup of cases in the logMAR range >2.0 is explained by the shift from visual acuity from a level measurable by chart to a visual acuity at the counting fingers level and below.

Best corrected visual acuity was correlated with refractive error (Fig. 2). Starting at the extreme myopic end of the curve, mean BCVA increased to 0.00 logMAR (or 1.0 decimal) for emmetropia, and decreased towards the high hyperopic end of the curve. Excluding eyes with a refractive error of less than −5 diopters or of more than +5 diopters led to a similar curve. Dividing the whole study population in a highly myopic group with a myopic refractive error of ≥−8 diopters, a moderately myopic group (myopic refractive error >−0.50 diopters and ≤−8 diopters), an emmetropic group (−0.5 diopters to +0.50 diopters), a moderately hyperopic group (>+0.50 diopters to ≤+4 diopters), and a highly hyperopic group (>+4 diopters) showed, that BCVA was significantly the highest in the moderately hyperopic group (0.08±0.15 logMAR (0.87±0.21 decimals); *P*<0.001), followed by the emmetropic group (0.16±0.52 logMAR (0.89±0.31 decimals), in which BCVA was significantly (*P*<0.001) higher than in moderately myopic group (0.28±0.33 logMAR (0.64±0.34 decimals), in which it was significantly (*P*<0.001) better than in the highly hyperopic group (0.66±0.62 logMAR (0.36±0.29 decimals), in which it was better (*P* = 0.002) than in the highly myopic group (1.32±0.92 logMAR (0.17±0.18 decimals)).

**Figure 3:**
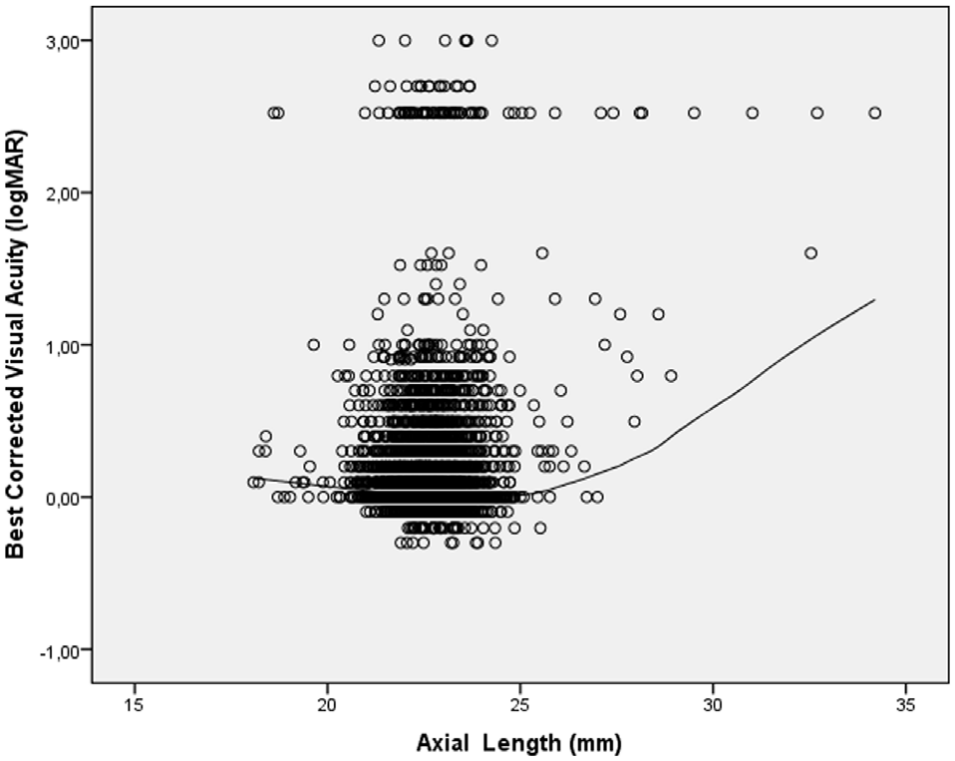
Scattergram showing the correlation between best corrected visual acuity (expressed as the logarithm of the minimal angle of resolution, logMAR) and axial length in the Central India Eye and Medical Study; the regression line was calculated using a locally weighted scatterplot smoothing (LOWESS) function. The subgroup of cases in the logMAR range >2.0 is explained by the shift from visual acuity from a level measurable by chart to a visual acuity at the counting fingers level and below.

Parallel to the correlation between BCVA and refractive error, BCVA was significantly associated with axial length. Starting at the high axial length end, the BCVA increased towards an axial length of about 23 mm, and then decreased again towards the shorter axial length end (Fig. 3). To assess a potential association between BCVA and axial length, the whole study population was stratified by axial length. The subgroups included cases with an axial length of less than <21.66 mm (representing 10% of all study participants), cases with an axial length of ≥21.66 mm and <22.42 mm (representing 30% of all study participants), cases with an axial length of ≥22.42 mm and <22.79 mm (representing 20% of all study participants), cases with an axial length of ≥22.79 mm and <25.90 mm (representing 39.5% of cases), and cases with an axial length >26.0 mm (representing 0.5% of all study participants. BCVA was significantly (*P* = 0.03 and *P* = 0.02, respectively) higher in the subgroup with an axial length of 21.66–22.42 mm (0.14±0.37 logMAR) and in the subgroup with an axial length of 22.43–22.79 mm (0.14±0.37 logMAR) than in the subgroup with an axial length of 22.80–25.90 mm (0.18±0.44 logMAR), in which it was significantly (*P*<0.001) better than in the subgroup with an axial length exceeding 26.0 mm (1.31±0.96 logMAR) and the subgroup with an axial length of less than 21.66 mm (0.18±0.40 logMAR; *P*<0.001). The two first mentioned subgroups did not differ significantly in BCVA (*P* = 0.95). BCVA was significantly (*P*<0.001) better in the group with short axial length (<21.66 mm) than in group with the high axial length (>26.0 mm).

**Figure 4:**
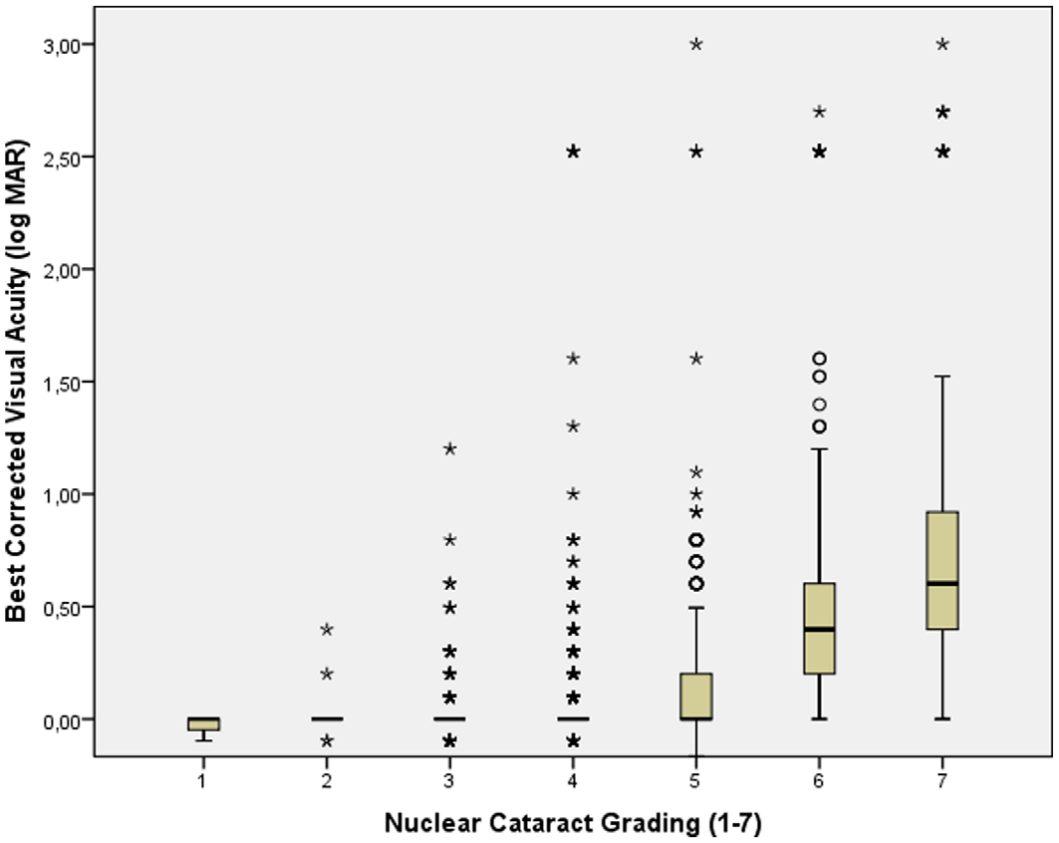
Scattergram showing the correlation between best corrected visual acuity (expressed as the logarithm of the minimal angle of resolution, logMAR) and the amount of nuclear cataract in the Central India Eye and Medical Study. The box contains the middle half of the study population, with the median (black horizontal line in the box) indicating the numerical value separating the higher half of the whole study population from its lower half. The end of the whiskers superior and inferior to each box represent the highest and lowest, respectively, value within a range of 1.5 times of the box length. The black dots represent the outliers which by definition are located between 1.5 to 3 times the box lengths (interquartile range) outside of the box. The asterisks represent extremes which by definition are located more than 3 times the box length (interquartile range) outside of the box. The subgroup of cases in the logMAR range >2.0 is explained by the shift from visual acuity from a level measurable by chart to a visual acuity at the counting fingers level and below.

**Figure 5:**
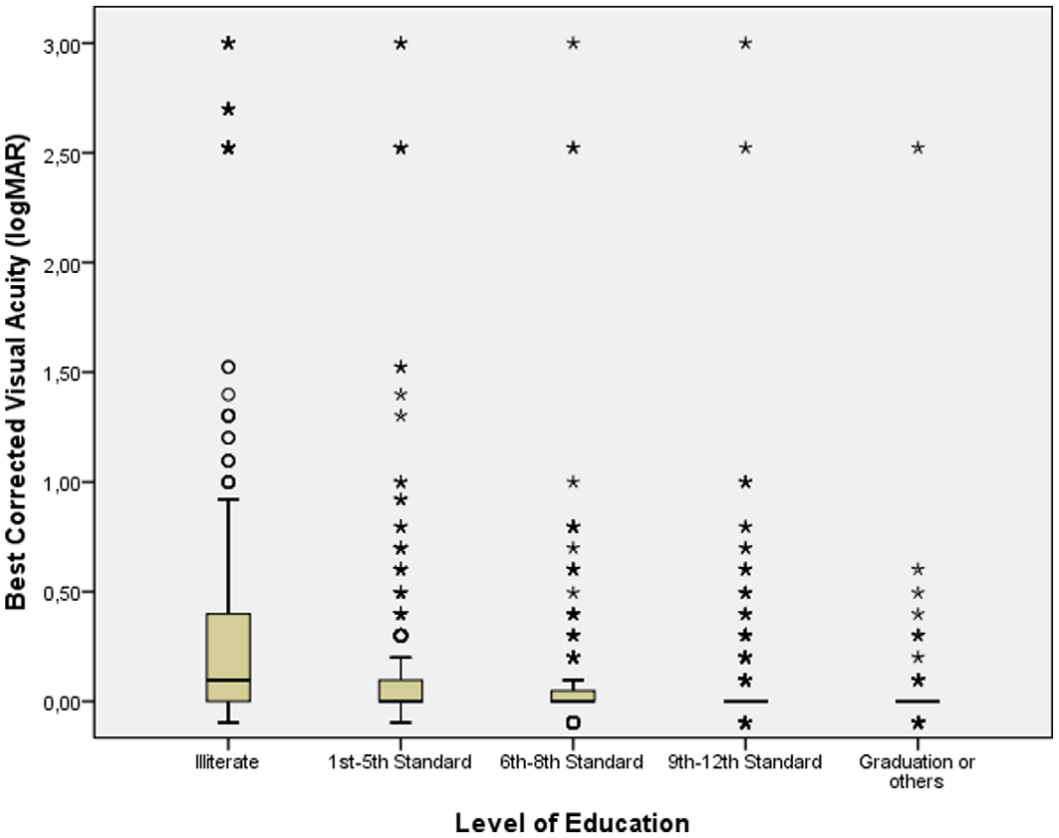
Scattergram showing the correlation between best corrected visual acuity and the level of education in the Central India Eye and Medical Study. The box contains the middle half of the study population, with the median (black horizontal line in the box) indicating the numerical value separating the higher half of the whole study population from its lower half. The end of the whiskers superior and inferior to each box represent the highest and lowest, respectively, value within a range of 1.5 times of the box length. The black dots represent the outliers which by definition are located between 1.5 to 3 times the box lengths (interquartile range) outside of the box. The asterisks represent extremes which by definition are located more than 3 times the box length (interquartile range) outside of the box. The subgroup of cases in the logMAR range >2.0 is explained by the shift from visual acuity from a level measurable by chart to a visual acuity at the counting fingers level and below.

**Figure 6:**
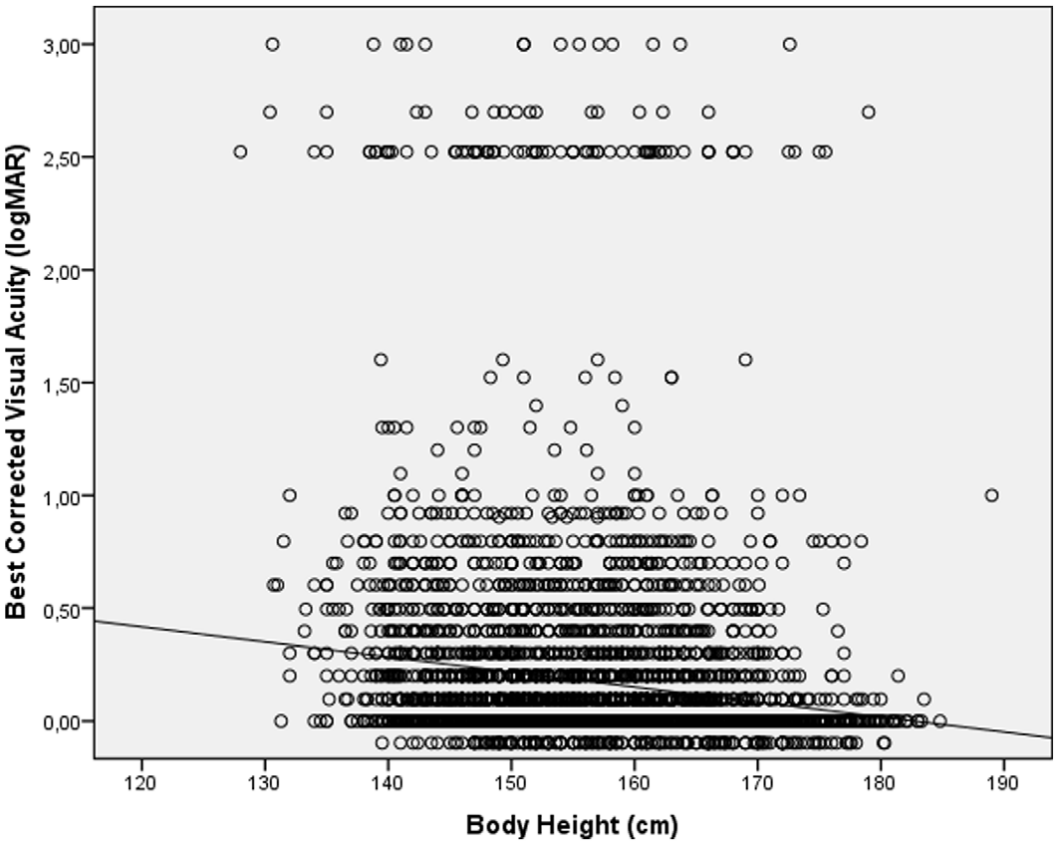
Scattergram showing the correlation between best corrected visual acuity and body height in the Central India Eye and Medical Study. The subgroup of cases in the logMAR range >2.0 is explained by the shift from visual acuity from a level measurable by chart to a visual acuity at the counting fingers level and below.

BCVA significantly worsened with the amount of astigmatic (cylindrical) refractive error (r = −0.35; *P*<0.001), age (r = −0.63; *P*<0.001), increasing amount of nuclear cataract (r = −0.61; *P*<0.001) (Fig. 4) and increasing intraocular pressure (r = −0.039; *P* = 0.008). BCVA improved significantly with the level of education (r = 0.36; *P*<0.001) (Fig. 5) and body height (r = 0.21; *P*<0.001) (Fig. 6), body weight (r = 0.26; *P*<0.001), and body mass index (r = 0.17; *P*<0.001). BCVA was not significantly different between men and women (*P* = 0.39).

### Multiple regression analysis

Since in univariate analysis, best corrected visual acuity was significantly associated with age, refractive error, level of educational, degree of nuclear cataract and cylindrical refractive error, and because some of these parameters such as degree of nuclear cataract and age were significantly (*P*<0.001) correlated with each other, a multiple regression analysis was performed. This multivariate analysis included the variables age, body height and body mass index, level of education, refractive error (spherical equivalent), axial length, degree of nuclear cataract and intraocular pressure as independent parameters. It revealed that the association with best corrected visual acuity was significant for the systemic parameters of lower age (*P*<0.001), higher level of education (*P*<0.001), higher body stature (*P*<0.001) and higher body mass index (*P*<0.001); and for the ophthalmic parameters of more hyperopic refractive error (spherical equivalent) (*P*<0.001), shorter axial length (*P*<0.001), lower degree of nuclear cataract (*P*<0.001), and lower intraocular pressure (*P* = 0.006) (Table 1).

**Table 1:** Results of the multivariate analysis of the associations between best corrected visual acuity (logMAR) as dependent parameter and ocular and general variables as independent parameters in the Central India Eye and Medical Study.

	*P*-value	Coefficient Beta	Regression Coefficient	95% CI
Age (Years)	<0.001	0.20	0.006	0.003, 0.009
Level of Education (1–7)	<0.001	−0.043	−0.013	−0.021, −0.004
Body Height (cm)	<0.001	−0.010	−0.004	−0.005, −0.003
Body Mass Index	<0.001	−0.045	−0.005	−0.008, −0.002
Refractive Error (Spherical	<0.001	−0.021	−0.044	−0.049, −0.038
Equivalent; Diopters))				
Axial Length (mm)	<0.001	0.013	0.055	0.043, 0.067
Nuclear Cataract (Grade 0–7)	<0.001	0.028	0.101	0.088, 0.113
Intraocular Pressure (mm Hg)	0.006	0.034	0.004	0.001, 0.007

P-Value: statistical significance of the association.

95%CI: 95% confidence interval for the coefficient.

## Discussion

Our study showed that the distribution of best corrected visual acuity in a rural population was associated with the systemic parameters of lower age (*P*<0.001), higher level of education (*P*<0.001), higher body stature (*P*<0.001) and higher body mass index (*P*<0.001), and with the ophthalmic parameters of emmetropia to slight hyperopia (*P*<0.001), lower cylindrical refractive error (*P* = 0.005), axial length between 21.66 mm and 22.79 mm (*P*<0.001), lower degree of nuclear cataract (*P*<0.001), and lower intraocular pressure (*P* = 0.006).

The results agree with previous investigations in that BCVA decreases with increasing age and advancing stage of cataract [2]–[5], [9], [10]. Relatively few studies reported an association between BCVA and a higher level of education. The Los Angeles Latino Eye Study LALES found that visual acuity was lower in individuals with less than 12 years of education [2]. In a similar manner, the Taiwanese Shihpai Eye Study reported a correlation between higher and higher education [11], [12].

Our study agrees with the Beijing Eye Study and the Taiwanese Shihpai Eye Study in that men and women did not differ in BCVA [10], [12]. It is in contrast to the Los Angeles Latino Eye Study LALES, the proyecto VER study on Mexican Americans in Arizona, the Salisbury Eye Evaluation Project in the USA, and the Australian Blue Mountains Eye Study and the Melbourne Visual Impairment Project, in which BCVA was significantly lower in women [2], [3], [13]–[16].

The associations between higher BCVA and higher body stature and higher body mass index have rarely been reported yet. Since the body mass index was significantly (*P*<0.001) associated with the level of education in our study population, and since in a similar manner, body height was significantly (*P*<0.001) associated with the level of education in our study population, the association between visual acuity and body mass index and body height may additionally reflect the correlation between visual acuity and the socioeconomic background. However, in populations more industrialized than the rural Central Indian population, the opposite association may be expected, since a higher body mass index is usually associated with a lower socioeconomic status in industrialized populations [17]. It is paralleled by recent reports on the association between body height and ocular parameters such as depth of the anterior chamber and prevalence of diseases such as angle-closure glaucoma [18]–[20].

The correlation between higher visual acuity and a range of refractive error between emmetropia to slight hyperopia agrees with studies on other ethnic groups in which myopia was associated with a lower visual acuity [1]–[5]. The finding of an association between lower visual acuity and higher intraocular pressure is due to the association between intraocular pressure and glaucomatous optic neuropathy.

Potential limitations of our study should be mentioned. A major concern in any prevalence study is nonparticipation. The Central India Eye and Medical Study had a reasonable response rate of 80.1%, but differences between participants and non-participants can lead to a selection bias. Second, subjects only from a markedly rural region were included, so that our study did not provide information on differences between rural and urban regions with respect to the examined parameters. Third, visual acuity measurements depend on the cooperation of the subjects. In view of the relatively low level of education, one may argue that some study participants did not fully understand their part of the examination technique. There was, however, plenty of time for the visual acuity measurement, since the study participants spent the whole day in the hospital for the study, and measurement of visual acuity was available for 99.9% of the study participants.

In conclusion, best corrected visual acuity in this rural population of Central India was associated with a higher socioeconomic background, higher body stature and higher body mass index. These findings were independent of the positive association to other systemic and ocular parameters such as lower age, emmetropia to slight hyperopia, axial length in the range from about 21.7 mm to 22.8 mm, lack of cataract, and lack of high intraocular pressure. In particular with respect to the association between visual acuity and the level of education, the question arises which of the two parameters influenced the other one.
